# Protein signatures of molecular pathways in non-small cell lung carcinoma (NSCLC): comparison of glycoproteomics and global proteomics

**DOI:** 10.1186/s12014-017-9166-9

**Published:** 2017-08-15

**Authors:** Shuang Yang, Lijun Chen, Daniel W. Chan, Qing Kay Li, Hui Zhang

**Affiliations:** Department of Pathology, Johns Hopkins Medicine, Smith Bldg 4013, 400 N. Broadway, Baltimore, MD 21287 USA

**Keywords:** Proteins, Glycoproteins, Non-small cell lung carcinoma (NSCLC), Squamous carcinoma (SqCC), Adenocarcinoma (ADC), Signaling pathway, Mass spectrometry (MS)

## Abstract

**Background:**

Non-small cell lung carcinoma (NSCLC) remains the leading cause of cancer deaths in the United States. More than half of NSCLC patients have clinical presentations with locally advanced or metastatic disease at the time of diagnosis. The large-scale genomic analysis of NSCLC has demonstrated that molecular alterations are substantially different between adenocarcinoma (ADC) and squamous cell carcinoma (SqCC). However, a comprehensive analysis of proteins and glycoproteins in different subtypes of NSCLC using advanced proteomic approaches has not yet been conducted.

**Methods:**

We applied mass spectrometry (MS) technology featuring proteomics and glycoproteomics to analyze six primary lung SqCCs and eleven ADCs, and we compared the expression level of proteins and glycoproteins in tumors using quantitative proteomics. Glycoproteins were analyzed by enrichment using a chemoenzymatic method, solid-phase extraction of glycopeptides, and quantified by iTRAQ-LC–MS/MS. Protein quantitation was further annotated via Ingenuity Pathway Analysis.

**Results:**

Over 6000 global proteins and 480 glycoproteins were quantitatively identified in both SqCC and ADC. ADC proteins (8337) consisted of enzymes (22.11%), kinases (5.11%), transcription factors (6.85%), transporters (6.79%), and peptidases (3.30%). SqCC proteins (6967) had a very similar distribution. The identified glycoproteins, in order of relative abundance, included membrane (42%) and extracellular matrix (>33%) glycoproteins. Oncogene-coded proteins (82) increased 1.5-fold among 1047 oncogenes identified in ADC, while 124 proteins from SqCC were up-regulated in tumor tissues among a total of 827 proteins. We identified 680 and 563 tumor suppressor genes from ADC and SqCC, respectively.

**Conclusion:**

Our systematic analysis of proteins and glycoproteins demonstrates changes of protein and glycoprotein relative abundance in SqCC (TP53, U2AF1, and RXR) and in ADC (SMARCA4, NOTCH1, PTEN, and MST1). Among them, eleven glycoproteins were upregulated in both ADC and SqCC. Two glycoproteins (ELANE and IGFBP3) were only increased in SqCC, and six glycoproteins (ACAN, LAMC2, THBS1, LTBP1, PSAP and COL1A2) were increased in ADC. Ingenuity Pathway Analysis (IPA) showed that several crucial pathways were activated in SqCC and ADC tumor tissues.

**Electronic supplementary material:**

The online version of this article (doi:10.1186/s12014-017-9166-9) contains supplementary material, which is available to authorized users.

## Background

Comprehensive genomic profiling of primary non-small cell lung carcinoma (NSCLC) has identified mutations of multiple driver genes, especially oncogenes such as *AKT1*, *ALK*, *EGFR*, *ERBB2*, *KRAS*, *MET*, *NRAS*, BRAF, *PIK3CA*, *RET*, *ROS1,* and others [1, 2]. Based on these findings, several clinical trials have been implemented that target these molecular pathways [3–5]. For example, therapeutic treatments targeting tumors with *EGFR* alterations and *ALK* gene rearrangements have shown improved outcomes [6–8]. However, NSCLC continues to be the leading cause of cancer mortality, accounting for approximately 27% of all cancer deaths in the United States. In 2017 alone, it is estimated that over 222,500 patients will be diagnosed with NSCLC and more than 155,870 patients will die from the disease [9]. Thus, it is critical to understand the role of molecular alterations in NSCLC development, progression, and treatment susceptibility.

NSCLC is the most common morphological type of lung carcinoma (85–90%) and it consists of two major histological subtypes (ADC: 40–50%; SqCC: 25–30%) and several other subtypes (5%) [10]. The development and progression of a NSCLC tumor is a multistep process. NSCLC tumors are characterized by aberrant gene and protein expression, which subsequently leads to phenotypic transformation of cells, initiation and progression of the tumor [6–8, 11]. The large-scale genomic analysis of NSCLC has demonstrated that molecular alterations are substantially different between ADC and SqCC [12, 13]. The alterations of *EGFR* and rearrangement of *ALK* in ADC are detected in approximately 25% of tumors; loss-of-function mutations in *LKB1/STK11*, *NF1*, *CDKN2A*, *SMARCA4* and *KEAP1* are also identified [12]. In contrast, SqCCs rarely harbor *EGFR* mutations or *AKL* rearrangement; instead, SqCCs demonstrate alterations in other genes such as *RTKs*, *DDR2* and *FGGRs*, and inactivated *CDKN2A, PTEN, KEAP1, MLL2, HLA*-*A, NFE2L2, NOTCH1* and *RB1* [13].

The complex alterations of genetic pathways are associated with aberrant cellular protein expression patterns. More than 50% of cellular proteins, including secreted, cell surface and intracellular proteins, are glycosylated. Protein glycosylation is known to play critical roles in the regulation of cell growth, differentiation and migration [14–16]. Glycoprotein expression may directly reflect the physiological and/or pathological status of the lung parenchyma. Glycosylation in NSCLCs occurs on diverse molecules and involves a large number of genetic and proteomic alterations; thus a single protein biomarker is unlikely to be representative of all NSCLCs. The profiling of proteins and glycoproteins is particularly important for understanding NSCLC biology and identifying candidate molecular markers.

Over the past decade, efforts have been devoted to the identification of protein biomarker candidates in the various forms of lung cancers. For example, more than six hundred articles have been published for predictive lung cancer biomarkers, while over three hundreds publications are related to prognostic biomarkers [17–20]. These observations demonstrate the general effort and interest in the discovery of potential protein biomarkers for detecting and monitoring the progression of lung cancer. Most of these studies used non-human tissues or body fluid to study genes or proteins; however, few have been focused on the glycoproteins in human lung tissues using hydrazide chemistry to specifically study protein glycosylation. Additionally, the molecular complexity of NSCLC is still not fully understood.

In this study, we focused on profiling the protein and glycoprotein signatures of primary lung SqCC and ADC using advanced proteomics and MS technology, and we compared glycoproteins and proteins in tumor tissues using Ingenuity Pathway Analysis (IPA) (http://www.ingenuity.com/products/ipa). The purposes of this study are: (1) to profile proteins and glycoproteins in two NSCLC subtypes; (2) to understand their potential roles in molecular signaling pathways; (3) to correlate signature proteins with cellular biological functions and tumor biological pathways, essentially for the discovery of molecular markers; and (4) to provide information regarding the potential indirect molecular targets in several well-known genetic pathways.

## Results and discussion

### Protein distribution in lung tissue

**Fig. 1:**
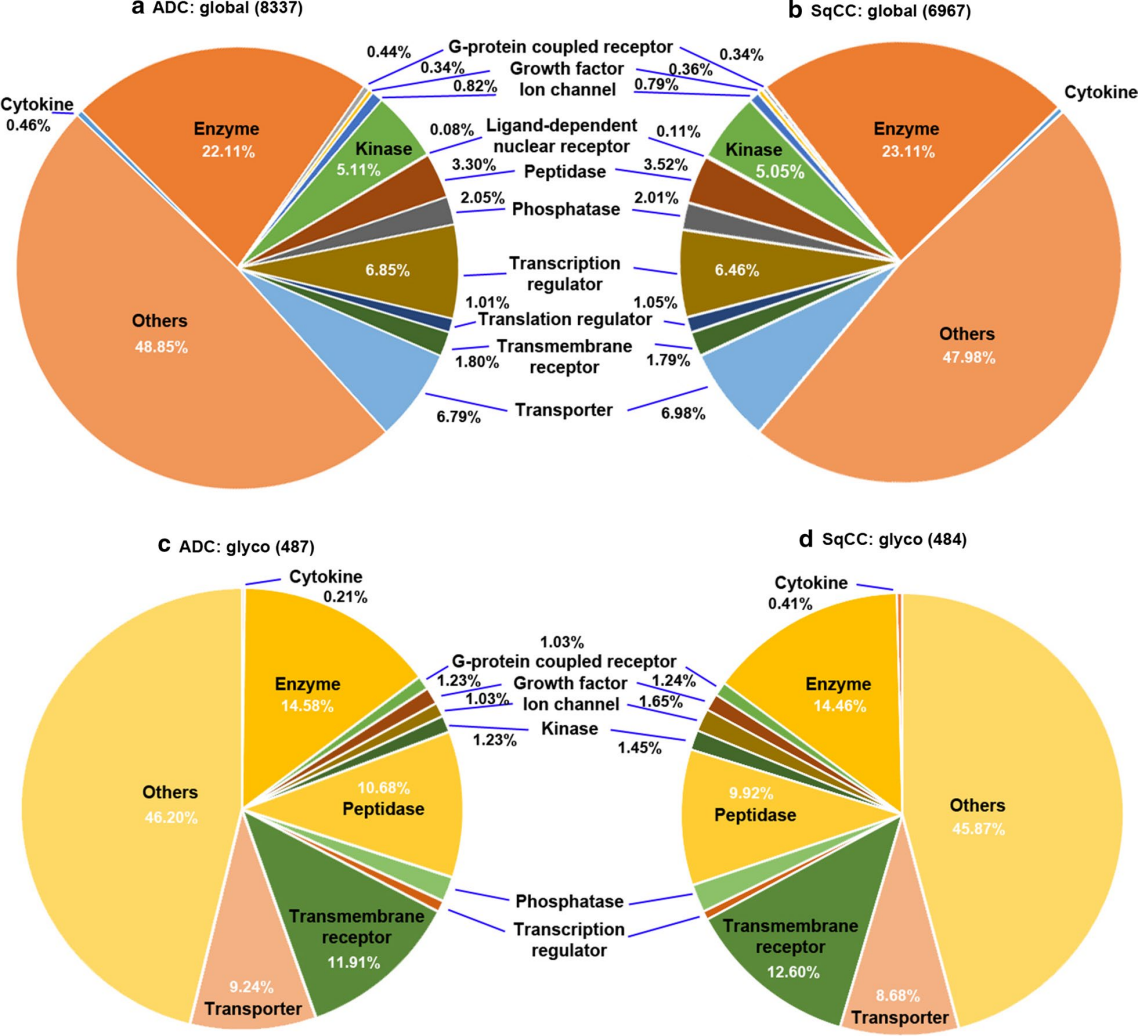
Functional distribution of global and glycoproteins identified from healthy control, normal tissues, lung SqCC, and ADC tissues. The proteins are classified based on their cellular functions, including enzyme, ion channel, kinase, peptidase, phosphatase, transcription regulator, transporter. **a** Global proteins in ADC, **b** global proteins in SqCC, **c** glycoproteins in ADC, and **d** glycoproteins in SqCC

**Fig. 2:**
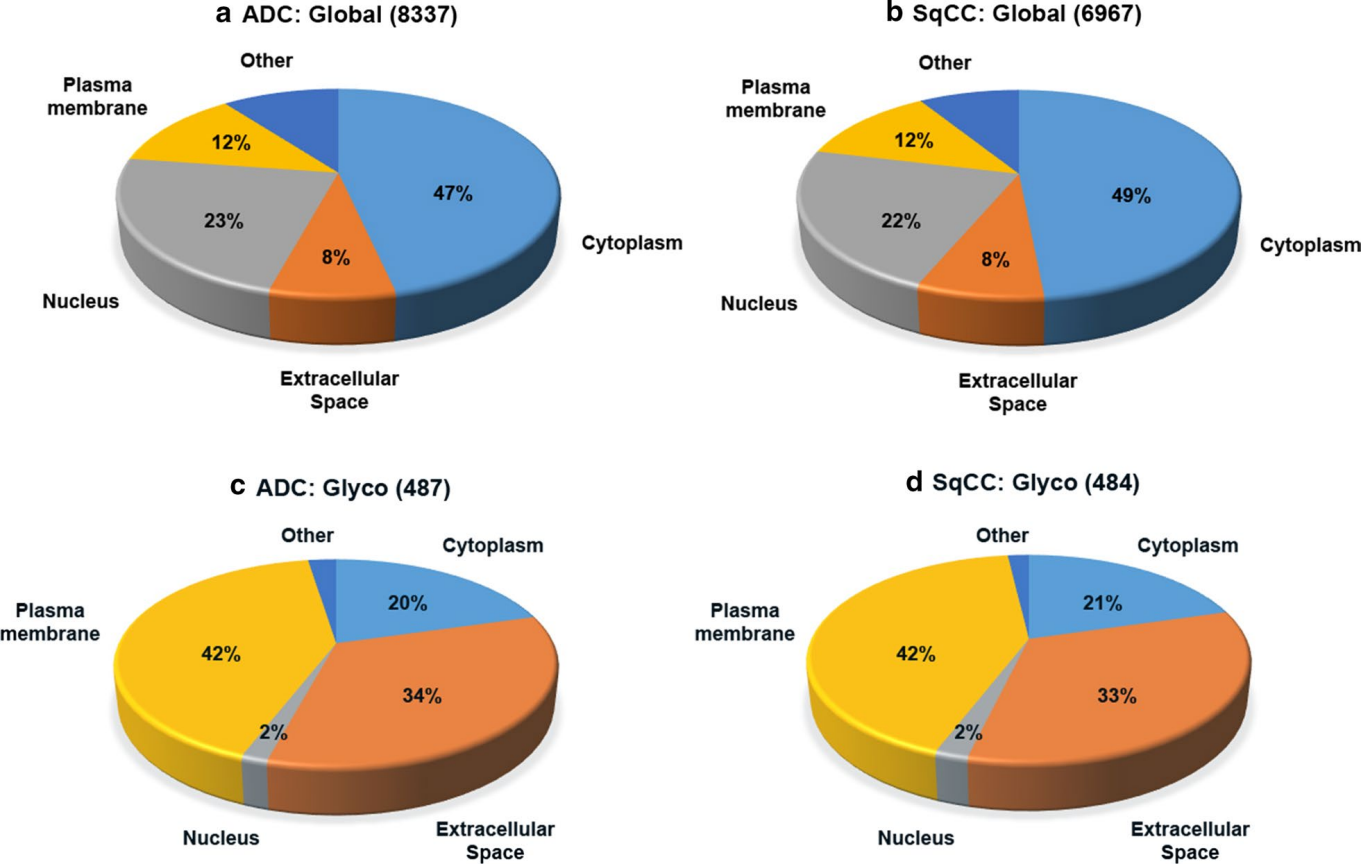
Cellular components of global and glycoproteins identified from lung SqCC and ADC. The major cellular components are cytoplasm, extracellular space, nucleus, and plasma membrane. **a** Global proteins in ADC, **b** global proteins in SqCC, **c** glycoproteins in ADC, and **d** glycoproteins in SqCC

Comprehensive profiling of proteins was performed on 18 lung tissues from normal, healthy control, ADC and SqCC patients. Over 8000 proteins were quantitatively identified in ADC (Fig. 1a) and 6900 proteins in SqCC (Fig. 1b). Many proteins were substantially increased in ADC and SqCC tumor tissues compared to the normal tissues, as shown in Additional file 2: Table S2, Additional file 3: Table S3, Additional file 4: Table S4, Additional file 5: Table S5. Proteins from ADC or SqCC were identically distributed based on their cellular types, and enzymes, transcription factors, transporters, kinases, peptidases, and phosphatases were dominant. Classification based on cellular location (Fig. 2) indicated that half of the proteins were cytoplasmic (47% in ADC and 49% in SqCC). The majority of glycoproteins were localized to the plasma membrane (42%), extracellular space (~33%), and cytoplasm (~20%) (Fig. 2c, d). Over 1000 enzymes were concurrently identified in the lung tissues: 241 proteins that are encoded by transcriptional factors were identified in both subtypes of NSCLC tissues. In lung tissue, about 5% of the proteins were kinases: 5.11% in ADC and 5.05% in SqCC. Among the 228 identified kinases in SqCC tissues, 33 of them were upregulated (>1.5-fold); only 3 kinases were slightly over-expressed and 4 were down-regulated in ADC out of a total of 248 kinases. Most kinases remained stable between 0.67-fold and 1.5-fold.

### Quantitative analysis of glycoproteins

We identified over 480 glycoproteins from lung ADC or SqCC tissues; 443 glycoproteins were present in both subtypes (Fig. 1c, d; Additional file 6: Table S6). Glycoproteins, such as DSC3, DSG3, PLOD2, DSC2, VCAN, PLOD1, DSG2, SLC2A1, TIMP1, and EGFR were increased in SqCC. Conversely, glycoproteins including PLOD2, DSG2, PLOD1, DSC2, and TIMP1 were up-regulated in ADC tumor tissues. Other glycoproteins were only found to be upregulated in ADC, including FAP, CALU, POSTN, and CEACAM6. The global results showed that CEACAM and MUC were significantly increased in ADC, suggesting that the upstream regulators of those glycoproteins may be strikingly upregulated different from SqCC. However, it is difficult to draw a conclusion only based on the similarity of a single protein or glycoprotein between ADC and SqCC. Instead, a panel of proteins or glycoproteins may be better than an individual entity to represent the diseases. A systematic analysis of protein activation was inferred through statistical interpretation using IPA [21, 22].

### Enzyme regulation

Enzymes are known to catalyze thousands of biochemical reactions and they are indispensable in the functions of living organisms. Proteins functioning as enzymes in the lung may regulate their biological functions and activate or inhibit diseases. Among them, the abundance of six proteins were substantially increased in both NSCLC subtypes, including BCAT1, UPP1, CARS2, HAT1, CD38, and PSAT1 (Note: protein name is given in the SI). Upregulated in both ADC and SqCC tissues, BCAT1 has been found to promote cell proliferation through amino acid catabolism in the malignant tumor of the glial tissue [23], whereas UPP1 along with other genes are predominantly expressed in the pancreatic ductal epithelium [24]. HAT1, CARS2, CD38, and PSAT1 are upregulated in SqCC but downregulated in ADC. A critical oncogene with roles in protein acetylation, HAT1 has been linked to different types of cancers. The CD38 protein is a marker of cell activation and it is associated with leukemia, myelomas, and solid tumors [25]. Overexpression of PSTA1 can stimulate cell growth and increase the chemoresistance of colon cancer cells [26]. Differential expression of these proteins may trigger varied phenotypes in NSCLC.

The relative abundance of other proteins, such as enzymes, have been exclusively changed in ADC or SqCC. Approximately 40 enzymes are upregulated in ADC tissues, notably ENPP2, LAMB2, TAB1, ASAH1, LAMP2, GPNMB, HSPG2, CTBS, GLA, and MAN1A1. ENPP2 is responsible for the production of lysophosphatidic acid from lysophosphatidylcholine. It has been identified as a potent tumor mitogen, a cell motility stimulating factor, and it plays a role in cell proliferation [27]. Recent studies indicated that overexpression of the ENPP2 gene increases cell tumorigenesis, invasion, and metastases in breast cancer [28]. Inhibition of ENPP2 can delay breast tumor growth and lung metastasis [29]. TAB1 (TGF-beta activated kinase 1) can mediate diverse intracellular signaling pathways, particularly the promotion of TGF-β mediated nuclear factor- κB (NFκB) activation during cancer progression [30]. Several matrix degrading enzymes (MMPs) were down-regulated in lung ADC tissues, resulting in the overexpression of extracellular matrix glycoproteins. An extracellular matrix glycoprotein, Laminin (LAMB2) contains the major non-collagenous constituent of basement membranes. This glycoprotein is involved in many biological processes including cell adhesion, signaling, differentiation, and metastasis [31]. A glycosylated beta subunit, ASAH1 cleaves a mature enzyme post-translationally, whose expression is correlated with improved prognosis in estrogen receptor-positive breast cancer [32]. LAMP2, GPNMB, and HSPG2 are associated with tumor cell metastasis and tumor growth [33–35]. Other genes can regulate protein glycosylation, e.g., degradation of asparagine-linked glycans (CTBS) [36], hydrolysis of the terminal alpha-galactosyl moieties of glycoproteins or glycolipids (GLA) [37], or catalysis of the hydrolysis of the three terminal mannose residues of N-glycans (MAN1A1) [38].

**Table 1:** Comparison of oncogenes and tumor suppressor genes (TSGs) on lung SqCC and ADC tumors

Gene	SqCC		ADC		Type
	mRNA, microRNA, and DNA sequencing [12]	Proteomics glycoproteomics	mRNA, microRNA, and DNA sequencing [13]	Proteomics glycoproteomics	
TP53	+	+	+	NE	Oncogene
KRAS	+	−		−	Oncogene
KEAP1	+	NC	+	ND	Oncogene
STK11	+	ND		−	TSG
EGFR	+	+		+	Oncogene
NF1	+	ND		ND	TSG
BRAF	+	NC		NC	Oncogene
SETD2	+	ND		ND	TSG
RBM10	+	NC		NC	TSG
MGA	+	ND		ND	TSG
MET	+	+		+	Oncogene
ARID1A	+	NC		NC	TSG
PIK3CA	+	Y−	+	−	Oncogene
SMARCA4	+	NC		−	TSG
RB1	+	NC	+	NC	Oncogene
CDKN2A	+	+	+	+	TSG
U2AF1	+	+		Y−	Oncogene
RIT1	+	−		NC	TSG
NOTCH1		ND	+	−	TSG
PTEN		NC	+	−	TSG
HLA-A*		+	+	+	Oncogene
NFE2L2		ND	+	ND	Oncogene
MLL2		ND	+	ND	TSG
FHIT		NC		NC	Oncogene
MST1		NC		−	TSG
BAD		NC		NC	Oncogene
RXR		+		ND	Oncogene
NFκBIA		−		−	TSG

The activation or inhibition is based on quantitative data from proteins and glycoproteins. The analysis was performed using Ingenuity Pathway Analysis (IPA). *NC* no change, *ND* not detected, *NE* no effect, “+” activation or upregulation, “−” inhibition or downregulation, “*” only detected in glycoproteins by SPEG. Gene mutation is compared with studies using mRNA, microRNA, and DNA sequencing on ADC [12] and SqCC [13]

### Transcriptional regulators

Some transcriptional regulators were substantially decreased (AGAP3, ACTN1, and TSC22D4), while FOXK1 was increased in ADC. An actin binding protein, ACTN1 cytoskeletal isoform is involved in binding actin to the membrane, and reduction of ACTN1 by siRNA can enhance tumor-free survival [39]. Conversely, other transcriptional regulators were increased in lung SqCC tissues, e.g., BTF3, PYCARD, NFκBIE, EDF1, HMGA1, MAX, MTDH, NMI, and LPXN. A transcription factor and a modulator of apoptosis, BTF3 (basic transcription factor 3) can initiate apoptosis and activate NFκB [40]. BTF3 interacts with CARD domain containing proteins such as PYCARD that mediate the assembly of apoptotic signaling complexes, leading to NFκB activation and increased protein expression [41]. EDF1, endothelial differentiation-related factor-1, is an important gene for tissue angiogenesis and cell proliferation [42]. The overexpression of HMGA1 could associate with the metastatic progression of NSCLC cells. In fact, HMGA1 is a non-histone protein that alters chromatin structure and regulates other transcriptional genes by either enhancing or suppressing transcription factors, exemplifying inhibition of the function of p53 family members in thyroid cancer cells [43, 44]. Functioning as an oncogene in many cancers and highly expressed in cancers, MTDH assists in cancer progression and development. It is induced by the c-MYC oncogene and plays roles in the anchorage-independent growth of cancer cells [45]. MAX, on the other hand, can form a dimer with c-MYC to promote cancer cell proliferation and normal cell apoptosis [46].

Several transcriptional proteins were detected only in either SqCC (32) or ADC (53) tissues. However, most proteins had negligible regulation (0.67–1.50-fold change) in either lung subtype, except for JUNB and ANKLE2 proteins. JUNB is uniquely overexpressed in ADC and it is involved in regulating gene activity following the primary factor response. JUNB can promote cell invasion and angiogenesis in cancer cell carcinoma [47]. ANKLE2 was upregulated only in SqCC (1.5-fold), indicating the unique characteristic of this transcriptional protein in SqCC.

### Protein kinases

Kinases transfer the phosphate groups from high-energy, phosphate-donating molecules to specific substrates, or vice versa. Protein phosphorylation or de-phosphorylation can greatly affect kinase activity, reactivity, and ability to bind other molecules. Kinases are thus essential in metabolism, cell signaling and other cellular pathways. Many protein kinases play roles in cell metabolism, including NADK, PKM, NAGK, PDK3, and ALDH18A1. Up-regulated in SqCC, AK4 (acetylate kinase-4; 2.98-fold) has been identified as a marker of poor clinical outcomes in NSCLC, and it can promote cancer metastasis via downregulation of the transcription factor ATF3 [48]. Other kinases have functions in the ERK, PI3K/Akt and PAK signaling pathways. For example, ZAP70 is a kinase required for association with the Shc adaptor protein and coupling of the activated TCR to the RAS/RAF/ERK signaling pathway [49]. Studies showed that the cross-talk between ERK and PI3K/Akt led to the intervention of cell cycle progression and cell death in carcinoma cells [50]. Overall, those proteins regulate diverse cellular pathways in cancer cell differentiation, transcription, proliferation, and apoptosis, e.g., MAP2K1, PRCKCB, PIK3CG, and TP53RK.

### Other highly regulated proteins

Major histocompatibility complex (MHC) proteins were over-expressed in both SqCC (MHC-DRBB1, MHC-B, and MHC-DQB1) and ADC (MHC-A, MHC-DRB1, MHC-C, MHC-DQB1, MHC-DRB3, and MHC-DQA1). Both subtypes of NSCLC have MHC class I and II. Importantly, the antigens of the MHC class I are associated with tumor growth and metastasis [51]. Loss of MHC class II gene and protein expression has been shown to be related to decreased tumor immune-surveillance and poor patient survival [52]. Collagens and extracellular matrix proteins are also highly expressed in SqCC (CTHRC1) and ADC (COL8A1, COL1A1, COL1A2). Over-expressed in ADC tissues only, CEACAM5 and MUC1 have been used as lung ADC markers, indicating that they are specific to this cancer subtype [53].

### Oncogenes and tumor suppressor genes

**Fig. 3:**
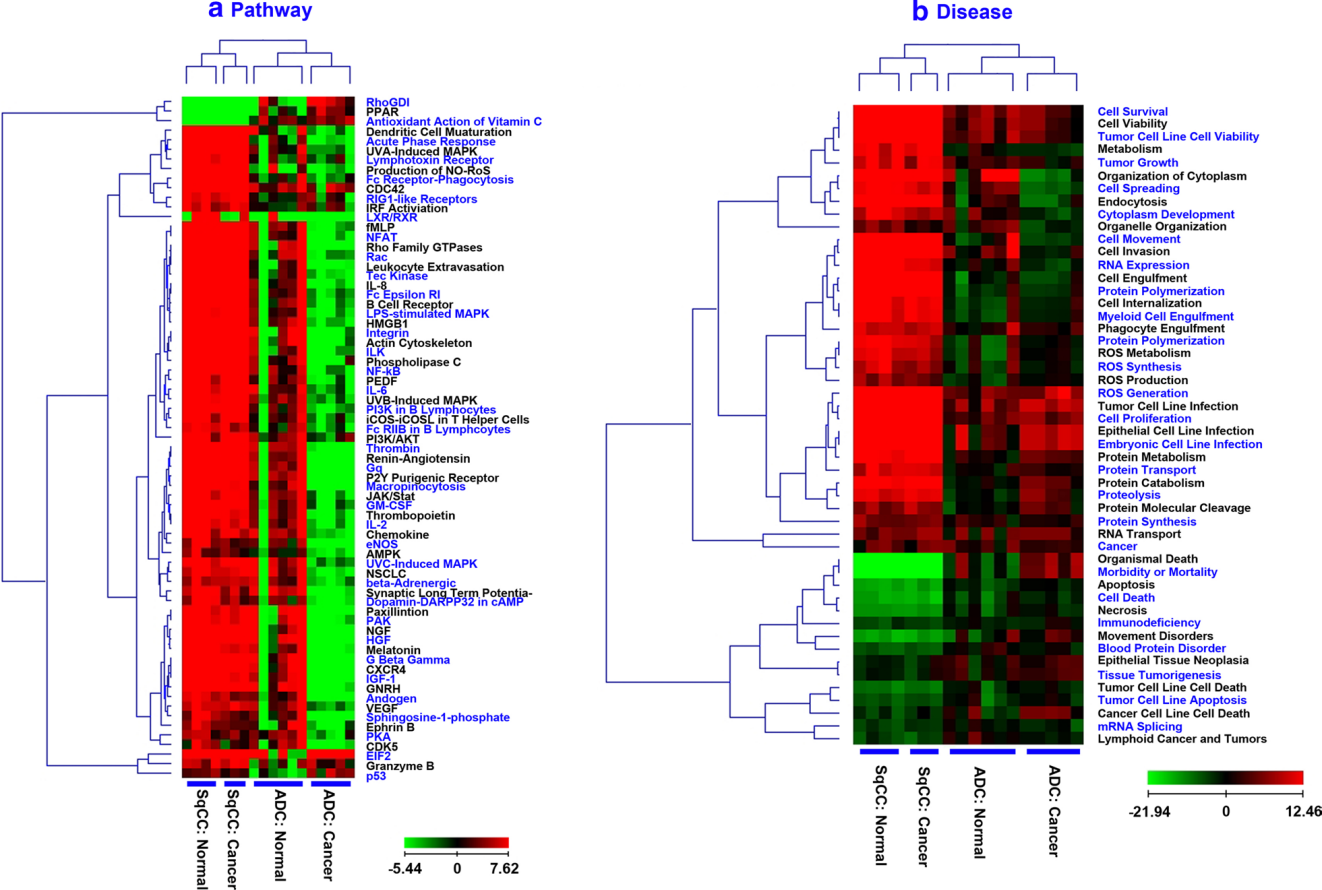
Heatmap of pathways and disease biological functions on lung SqCC and ADC. The individual specimen is used as a separate input and it is grouped for clustering. **a** Pathway comparison of SqCC and ADC (cancer vs. normal). The *positive values* indicate activation on the pathway, and vice versa. **b** Analysis of diseases and biological functions in SqCC and ADC (cancer vs. normal). The regulation in global proteins leads to activation or inhibition of each disease

Oncogenes and tumor suppressor genes (TSG) may be associated with the onset and progression of tumors. TSG or oncogene mutations could trigger a loss or reduction in cell functions, resulting in aberrant cell cycle progression. Also discussed previously, we found that many TSG-coded proteins are regulated in lung cancers [54–57]. Hence, it is reasonable to focus our analysis on those proteins whose genes are oncogenes or TSGs. As shown in Fig. 3A, we identified about 327 oncogenes (5%) and 563 TSGs (8%) in SqCC tissues, and 1047 oncogenes (12%) and 680 TSGs (8%) in ADC tissues (SI). There were 102 upregulated oncogene-coded proteins (>1.5-fold) and 46 down-regulated TSG-coded proteins (<0.67-fold); 82 oncogenes were overexpressed and 41 TSGs were down-regulated (Fig. 3A). These genes were further studied by pathway analysis to determine their relationship with transcription regulators and how they correlate to diseases (Fig. 3B, C).

Table 1 and Additional file 1: Table S1 list the upstream regulators in lung cancers and their regulated oncogenes/TSGs (IPA analysis). Three observations are evident: (a) activation in SqCC and ADC, (b) inhibition in SqCC and ADC, and (c) activation in only one of the subtypes. For example, ERBB2 is activated in both SqCC (z-score = 1.35) and ADC (z-score = 0.45); TP53 is inhibited in both lung cancer subtypes (−0.70; −2.32); and NFκBIA is inhibited in SqCC (−1.49) while it is activated in ADC (0.45). The upstream regulators (Additional file 1: Table S1) are plotted in Fig. 3Ba which compares major transcriptional regulators in SqCC and ADC. In general, most transcriptional regulators are activated in SqCC but inhibited in ADC. Further, we illustrated the downstream regulation of this transcription factor on oncogenes and TSGs (Fig. 3Bb–d). TP53 inhibition causes the differential regulation of genes in ADC and SqCC. For example, TP53 inhibition downregulates CAT, CAV1, CDKN1B, CNN1, CTGF, and CXCL12 in both ADC and SqCC (Fig. 3Bb); it increases the expression of TOP2A, COL1A2, and MCM2 in ADC, and it reduces the expression of ZYX, ANXA1, ASS1, CD82, DKK3, FAS, NDRG2, and PTPN11 in SqCC.

Protein expression may be related to a variety of diseases and biological functions in cells. Figure 3Ca lists the effect of protein regulation on different diseases and biological functions. The x-axis displays the disease-biological functions and the z-score is indicated on the y-axis. The diseases or biological functions are activated when Z > 0 and they are inhibited when Z < 0. To illustrate the results, a few examples are used for inversely regulated (Fig. 3Cb) and concurrently increased (Fig. 3Cc) biological functions. SqCC cytoplasm development is inhibited by a set of oncogenes (TNC, TACSTD2, KSR1, EGFR, CDK5) and TSGs (DMD, CXCL12, CDGF, CDKN1B, CDH13, CAVS, VIL1). ADC cytoplasm development is activated by oncogenes (JUNB, BOP1) and TSGs (ANXA1, CD44, PTPN11, TSC1, ZYX). The proliferation of tumor cells is activated by different sets of oncogenes and TSGs in both SqCC and ADC (Fig. 3Cc). Whether these genes are responsible for the specific diseases or biological functions needs to be further validated.

Oncogene- and TSG-coded proteins are important in lung SqCC and ADC. From the analysis of diseases, biological functions, and upstream regulators (Additional file 1: Table S1), SqCC may have signature oncogenes, e.g., EGFR, HMGA1, NFKB2, TNC, TFRC, CD274, and CDK5, and signature TSGs, e.g., CXCL12, CTGF, DCN, CDKN1B, and CAV1. Signature oncogenes in ADC are quite different and consist of JUNB, COL1A2, TOP2A, and ST6GAL1; TSGs include FAS, ARG1, CD44, GJA1, ITGA5, and ZYX. This panel of molecules may be useful for the diagnosis and prognosis of SqCC and ADC in lung cancers.

### NSCLC signaling in SqCC and ADC

**Fig. 4:**
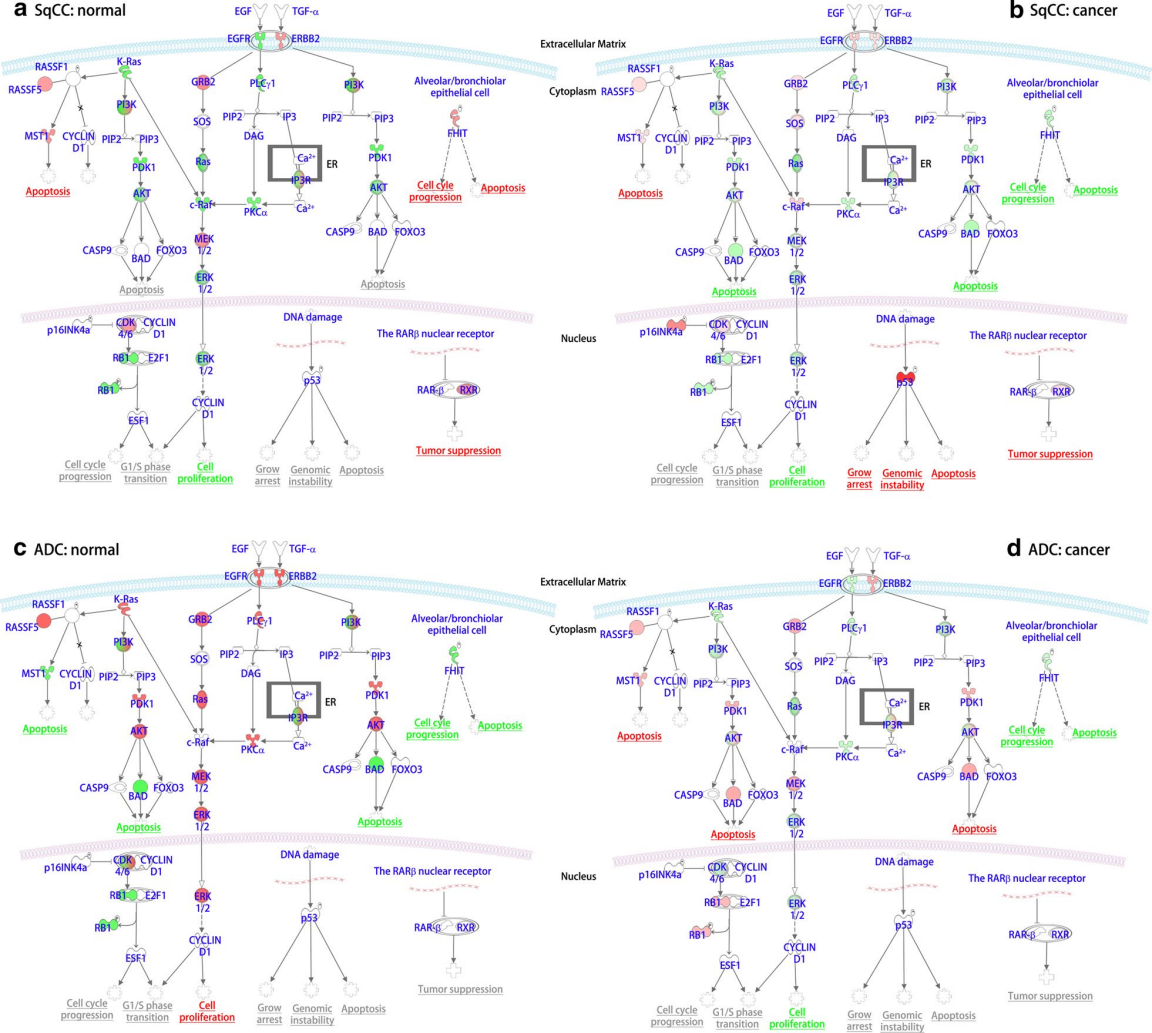
Comparison of non-small cell lung carcinoma (NSCLC) signaling pathways in SqCC and ADC. The NSCLC signaling pathways include enzymes, kinases, and transcriptional factors that have roles in protein expression and disease-related biological functions. **a** SqCC in normal tissue, **b** SqCC in cancer tissue, **c** ADC in normal tissue, and **d** ADC in cancer tissue. *Note: green color* shows inhibition and *red color* indicates activation of a cellular function

IPA analysis of proteins and glycoproteins reveals whether a signaling pathway is activated in SqCC and ADC. How the molecular markers are regulated in NSCLC signaling can be evaluated using proteins identified from SqCC and ADC (benign vs. cancer). Figure 4 shows the molecular markers that regulate the biological functions in lung cancers. The proteins are listed by their cellular location such as extracellular matrix, cytoplasm, and nucleus. In benign SqCC (Fig. 4a), EGFR (or HER1) is down-regulated and ERBB2 (or HER2) is upregulated by extracellular matrix proteins. EGFR downregulation leads to the overexpression of GRB2 (inhibition effect) or downregulation of PLCγ1 (activation effect). Together with an activation effect by K-Ras (down-regulated in SqCC normal), c-Raf is down-regulated and eventually inhibits cell proliferation. Although EGFR is upregulated in SqCC cancer, the same signaling and downstream regulation is observed, indicating that regulation of EGFR alone may not affect cell proliferation or apoptosis (Fig. 4a, b).

TSGs (TP53, FHIT) show dramatically different effects on cell growth arrest, genomic instability, and apoptosis (Fig. 4a, b). In SqCC normal tissue, p53 has no observable effect on cell diseases or functions. However, it is drastically activated in SqCC cancer, leading to the activation of cell apoptosis. Numerous studies have demonstrated that the increased expression of p53 mutation was associated with primary lung cancer [58, 59] and SqCC in the head and neck [60]. In contrast to p53, FHIT is overexpressed in normal SqCC, activating alveolar or bronchiolar epithelial cells. The deletion of this gene has been found in primary effusion lymphoma cell lines [61]. Along with K-Ras and PTEN, FHIT is genetically altered in ADC and SqCC tumors [62]. These results may indicate the importance of studying TSGs.

**Fig. 5:**
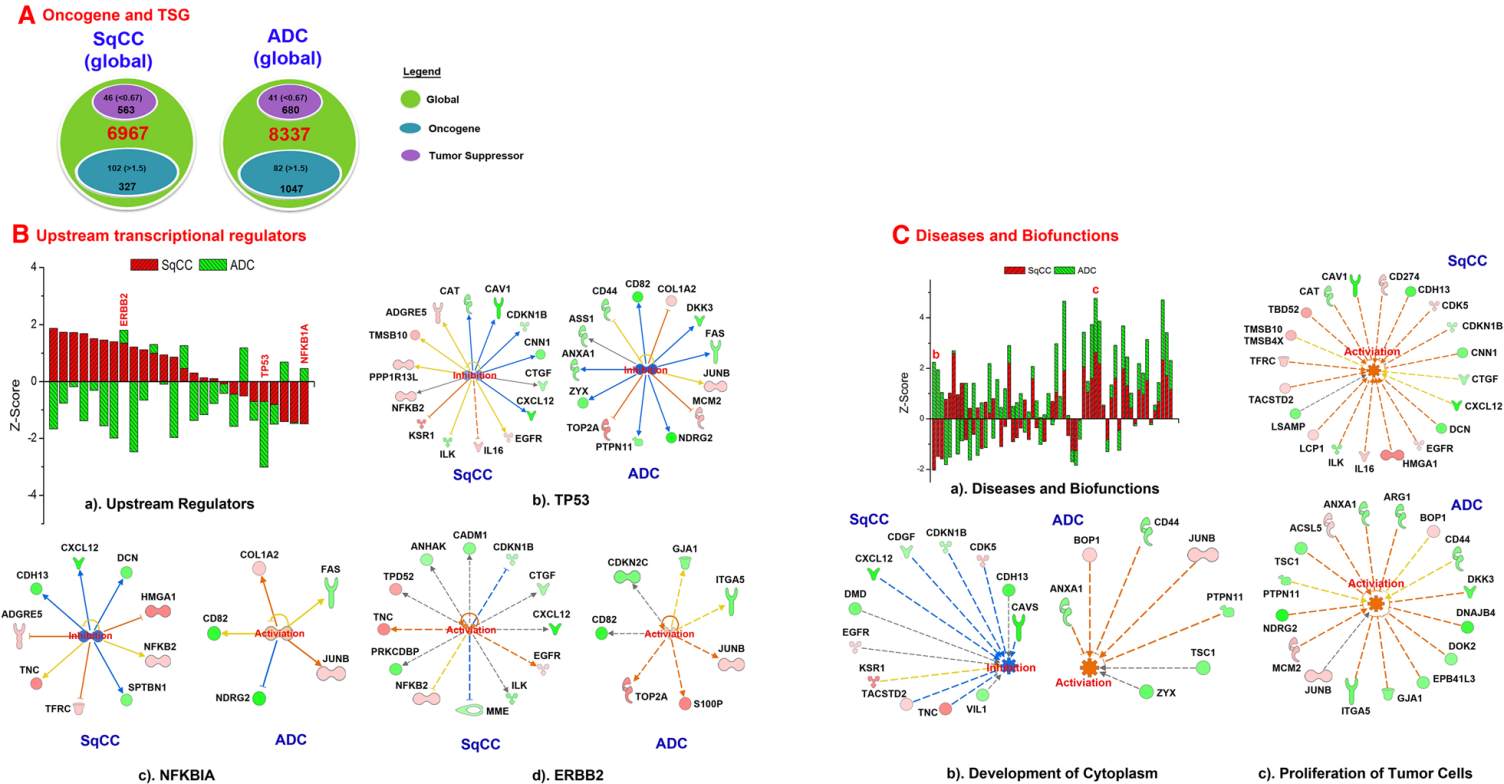
Quantitative analysis of oncogenes and tumor suppressor genes in SqCC and ADC. The oncogene and tumor suppressor gene (TSG) were obtained from the global data. **a** Distribution of oncogenes and TSG in SqCC and ADC, and **b** Upstream transcriptional regulators in SqCC and ADC. (*a*) Activated or inhibited oncogenes and TSGs, including TP53 (*b*), NFKBIA (*c*), and ERBB2 (*d*). **c** Distribution of diseases and biological functions in lung SqCC and ADC. (*a*) Activated or inhibited diseases or biological functions in SqCC and ADC, including development of cytoplasm (*b*), and proliferation of tumor cells (*c*)

 NSCLC signaling pathways in ADC tissues have differentially regulated protein signatures in response to changes of the extracellular matrix proteins. Different from SqCC, EGFR is overexpressed in normal ADC, but it is down-regulated in cancerous ADC (Fig. 5a, b). Most molecular signatures are increased in ADC normal tissues, potentially leading to cell proliferation, whereas the opposite phenomenon occurs in ADC tumor tissues (Fig. 5c, d). The overexpression of K-Ras in normal ADC also indirectly downregulates MST1, resulting in the increased incidence of apoptosis. However, MST1 is upregulated in ADC tumor through the reduced expression of K-Ras. Likewise, the highly increased expression of PDK1 and AKT in non-cancerous patients causes the downregulation of BAD, which further leads to decreased apoptosis. In ADC tumors, both MST1 and BAD are upregulated by their upstream regulators, resulting in increased apoptosis. Particularly, p53 remains stable in ADC; however, FHIT shows inhibition in alveolar/bronchiolar epithelial cells. Cell cycle progression and apoptosis are inhibited in ADC tissues.

RXR, retinoid X receptor, exhibits different effects in SqCC and ADC. RXT expression remains stable in ADC; however, the expression of RXR increases in SqCC. As a tumor suppressor, the increased RXR is expected to reduce tumor growth and progression. This result may suggest that the RXR gene may not be the dominant tumor suppressor gene in lung SqCC. Other tumor suppressors including p53 or FHIT may play dominant roles in lung SqCC.

### Regulation of protein glycosylation by oncogenes or TSGs

**Fig. 6:**
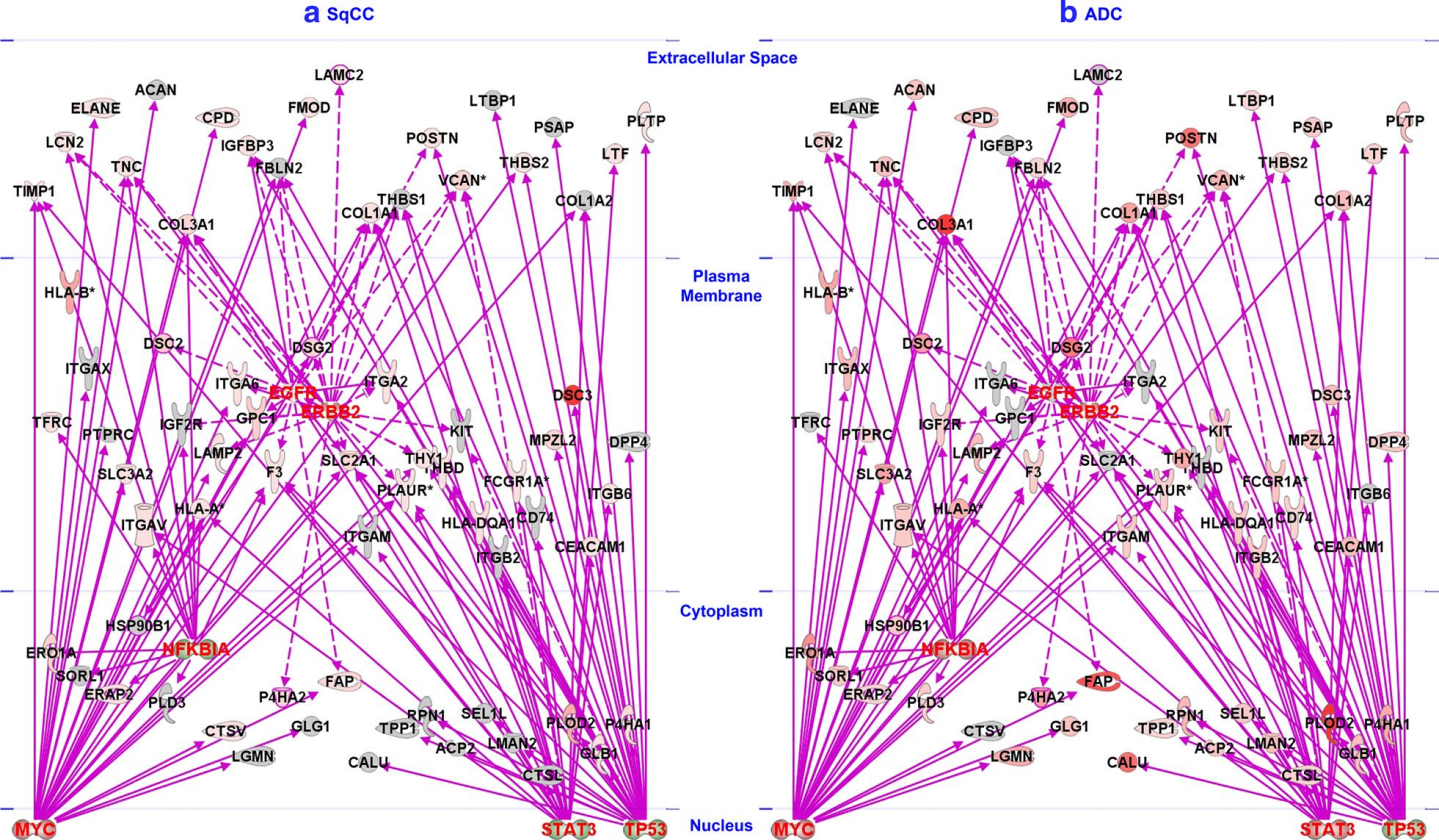
Glycoprotein regulation by transcriptional regulators, kinases, and enzymes. The gene networks were established using global and glycoprotein data via proteomics and Ingenuity Pathway Analysis. Glycoproteins that are regulated by transcriptional regulators (MYC, STAT3, TP53, and NFKBIA) and enzymes (EGFR and ERBB2) in SqCC (**a**) and ADC (**b**)

Mutation of oncogenes or TSGs can regulate protein glycosylation whose aberrant modification could associate with diseases. The oncogenes (Additional file 2: Table S2, Additional file 3: Table S3) or TSGs (Additional file 4: Table S4, Additional file 5: Table S5) that we identified in the current study might affect the expression of glycoproteins in SqCC or ADC. We studied the effect of several key proteins on glycoprotein expression, including oncogenes (ERBB2, MYC, and EGFR) and tumor suppressors (NFκBIA, STAT3, TP53). Figure 6 shows the regulation of glycoproteins by oncogenes or TSGs in SqCC and ADC. The results from the quantitative analysis of glycoproteins can be found in Additional file 6: Table S6. MYC and ERBB2 are activated in both SqCC and ADC, while TP53 is inhibited. EGFR is only activated in SqCC but it is inhibited in ADC. Similar patterns were observed for STAT3 and NFκBIA which were inhibited in SqCC and activated in ADC.

Glycoproteins in the extracellular matrix space are important for indicating diseases and they are likely secreted molecules in circulating body fluids [63]. These glycoproteins can be regulated by single or multiple transcriptional factors, kinases or enzymes. Despite different subtypes, extracellular glycoproteins such as TIMP1, LCN2, TNC, COL3A1, CPD, FOMOD, POSTN, VCAN, THBS2, LTF, and PLTP are useful for the identification of NSCLC or its specific subtypes. On the other hand, ELANE and IGFBP3 are only overexpressed in SqCC (Fig. 6a), while ACAN, LAMC2, THBS1, LTBP1, PSAP, and COL1A2 are uniquely upregulated in ADC. Other glycoproteins in the plasma membrane or cytoplasm are differentially regulated by transcriptional factors (MYC, STAT3, TP53, and NFκBIA). Interestingly, some glycoproteins in Fig. 6 are also oncogenes or tumor suppressors, such as TNC and COL1A2. TNC (tenascin-C) was overexpressed in NSCLC, leading to downregulation of the functions of infiltrating lymphocytes and implication in tumor progression [64]. Our results indicate that MYC activates TNC, but TP53 inhibits its expression (Fig. 6). In fact, TNC degradation is associated with tumor recurrence in early stage in NSCLC [65]. Collagen (COL1A2), together with other proteins that are overexpressed only in ADC, may be specific to ADC subtypes since it is deactivated in lung SqCC.

## Conclusion

Profiling of global proteins and glycoproteins could be an effective method for understanding molecular signaling pathways and the regulation of proteins that are associated with diseases and biological functions. Signature proteins may be useful for predicting disease onset and progression. Based on quantitative analysis of those proteins, we identified the corresponding genes that contain the instructions for producing the proteins. However, studies on genes alone do not necessarily shed light on how their proteins are post-translationally modified. Glycoprotein analysis can investigate beyond genomics and provide information regarding protein expression. Therefore, quantitative analysis of proteins and glycoproteins is essential for discovery of molecular markers.

In this study, the global proteins and glycoproteins from 18 lung tissues were analyzed. Over 6000 global proteins were quantified from both SqCC and ADC, and about 480 glycoproteins were identified from these tissues. Several proteins including enzymes, kinases, or transcription factors were overexpressed in SqCC and ADC, suggesting their common contributions in those subtypes. Many proteins are exclusively regulated in SqCC or ADC: ENPP2, LAMB2, TAB1, GPNMB, and FOXK1 are associated with ADC, while NFκBIE, EDF1, HMGA1, and LPXN are exclusively overexpressed in SqCC. Therefore, it is possible to use these genes to identify upstream regulators for targeted treatment, whereas the overexpressed glycoproteins can be further studied in lung bronchoalveolar lavage (BAL) for discovery of biomarkers for early detection. Further validation of these proteins can be used to identify a molecular panel for lung cancer, thereby increasing the accuracy of early stage detection.

Assuming that the protein level is proportional to gene expression, IPA analysis could determine the upstream regulators and identify the disease-associated proteins in NSCLC [21]. We have used protein data to correlate gene expression to the biological functions and diseases. Bioinformatics analysis of proteins and glycoproteins explores the essential relationship among proteins and glycoproteins.

## Experimental procedures

### Materials and reagents

All chemicals and reagents were purchased from Sigma Aldrich (St Louis, MO) unless specified otherwise. C18 Solid-phase extraction (SPE) cartridges (3 cc Vac Cartridge, 500 mg sorbent) were from Waters Corporation (Milford, MA). Peptide-*N*-glycosidase F (PNGase F) and denaturing buffer (10×) were from New England Biolabs (Ipswich, MA). Trypsin gold was from Promega (Madison, WI). Quantitative analysis of peptides was performed on a Q-Exactive mass spectrometer (Thermo Scientific, Waltham, MA). Human lung tissues were collected from the Department of Pathology with the approval of the Institutional Review Board of the Johns Hopkins University.

### Tissue protein analysis

**Fig. 7:**
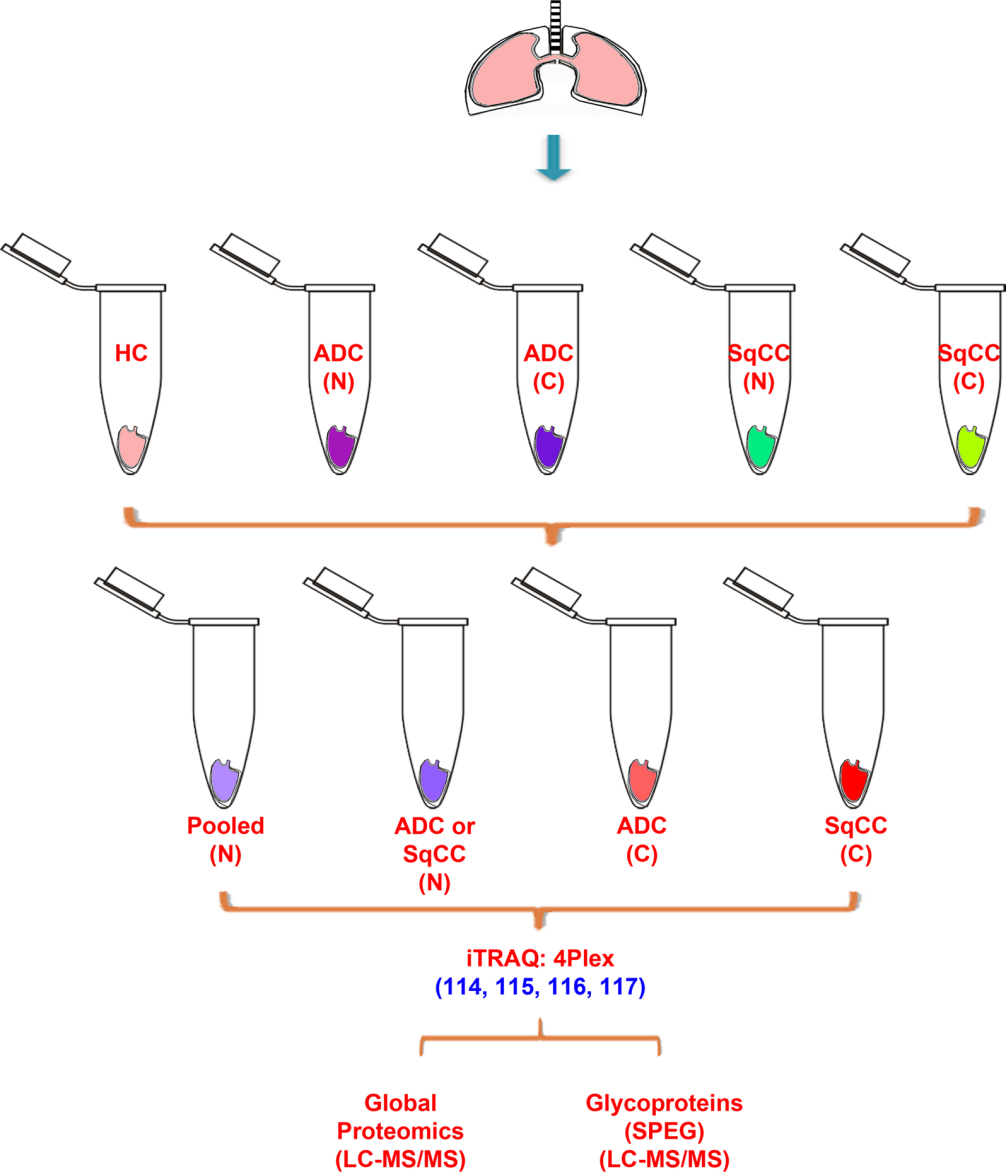
Schematic diagram of lung tissue specimens using for proteomic analysis. The dissected tissues in lysis buffer were sonicated to extract proteins. After 4-plex iTRAQ labeling, 10% of the peptides were analyzed for global proteins and 90% of the peptides were used for enrichment of glycosite-containing peptides by Solid-phase extraction of N-linked glycopeptides (SPEG)

A total of 18 patient tissues were collected, consisting of one normal tissue from a healthy control (HC), 3 cases of SqCC and tumor-matched benign tissues, and 6 cases of ADC and 5 tumor-matched benign tissues (Table 2). The collected tissues were dissected using razor blade before being sonicated in an ice-cooled bath for protein extraction [lysis buffer (1 mL): 1 M NH_4_HCO_3_ in the presence of 8 M urea] (Fig. 7). Protein concentration was measured by BCA assay (Pierce, Rockford, IL). Four mg proteins from each tissue were added to the final volume of 1 mL (8 M urea in 1 M NH_4_HCO_3_, pH 8.0) [63, 66]. Proteins were reduced in 10 mM of tris (2-carboxyethyl) phosphine hydrochloride (TCEP; 20 µL at 0.5 M) at 37°C for 1 h, followed by alkylation using 40 mM of iodoacetamide (IAA) for 1 h at room temperature in the dark. The same amount of TCEP (40 mM) was added to quench excess IAA. Samples were then diluted fivefold (volume) with DI water to contain 1.6 M urea for tryptic digestion (37°C, overnight; trypsin vs. protein = 1:40). The acidified peptides (~0.1% TFA) were cleanup by C18 SPE column (5×, 0.1% TFA) and eluted using 60% ACN (500 µL × 2, 0.1% TFA). Peptide concentration was determined using BCA and peptides were stored at −80 °C prior to use.

**Table 2:** Experimental design of tissues from human lung

List	ID	Age	Sex	Pathology	iTRAQ	iTRAQ group	Set	Position	Type	Dimension (mm)	Size of tumor
1	HC	68	M	HC		115	1				
2	SqCC.N1	80	M	N	114	116	1	LUL	SqCC	6.5	pT2b
3	SqCC.C1			C		117	1				
4	SqCC.N2	82	M	N	114	115	2	UL	SqCC	3	pT1b
5	SqCC.C2			C		116	2				
6	SqCC.N2	74	M	N	114	117	2	RUL	SqCC	4.7	pT2a
7	SqCC.C3			C		115	3				
8	ADC.N1	79	F	N		116	3	RUL	ADC	1.6	pT1
9	ADC.C1			C		117	3				
10	ADC.N2	74	F	N	114	115	4	RUL	ADC	3	pT1b
11	ADC.C2			C		116	4				
12	ADC.N3	65	M	N		117	4	RLL	ADC	3.3	pT2a
13	ADC.C3			C		115	5				
14	ADC.N4	71	M	N	114	116	5	RLL	ADC	5.2	pT2b
15	ADC.C4			C		117	5				
16	ADC.C5			C		116	6				
17	ADC.N5	61	F	N	114	115	6	RLL	ADC	4.5	pT3
18	ADC.C6			C		117	6				

Proteins from six benign tissue sections (N = normal) were pooled and labeled by iTRAQ channel 114. Proteins from 18 specimens including benign, health control and tumor tissues were labeled by 4-plex iTRAQ reagents (115, 116, and 117). The labeled proteins were pooled to six sets of iTRAQ groups. Proteins or glycoproteins were quantified by the relative intensity of 115, 116, 117 versus that of 114 in each group. Change of protein expression was estimated by iTRAQ quantitation. *RUL* right upper lung, *RLL* right left lung, *HC* health control, *N* normal (or benign), *C* cancer, *SqCC* squamous cell carcinoma, *ADC* adenocarcinoma. The HC was used as one of the controls for the determination of fold-change of proteins in cancer tissues

 One mg peptides from each sample were labeled with 4-plex iTRAQ (AB SCIEX, Framingham, MA). The iTRAQ labeled peptides were pooled for C18 cleanup and 10% (~200 µg) of the pooled peptides was chromatographically separated to 24 fractions by basic reverse phase liquid chromatography (bRPLC) on an 1220 Infinity LC system with a Zorbax Extend-C18 analytical column (1.8 µm particles, 4.6 × 100 mm; Agilent Technologies, Inc., CA) [66]. The remaining iTRAQ-peptides (~1.8 mg) were enriched for glycosite-containing peptides using hydrazide chemistry [67]. One µg of the enriched formerly glycosite-containing peptides were analyzed by LC–MS (Additional file 7, Additional file 8, Additional file 9, Additional file 10).

### MS data analysis

The MS/MS spectra were directly searched using the SEQUEST search engine [Thermo Proteome Discoverer 1.4.0.288 (PD)] against the NCBI Homo Sapiens database (Download, August 2014). Carbamidomethylation of cysteine residues was set as a fixed modification; oxidation of methionine and deamidation (only for SPEG) of asparagine were set as variable modifications; N-termini and lysines were set as iTRAQ 4-plex fixed modifications. Maximum missed cleavages using trypsin were set to 2 and the minimum peptide length was 7. The search filter was set as follows: at least 1 peptide per protein and 1% FDR for PSM cutoff. The precursor mass tolerance was 10 ppm, while the mass tolerance of fragment ions was 20 ppm. Quantitation was performed using reporter ion intensity of peptides in PD. The filter for PD was set as at least one peptide per protein and high confidence. The quantification was performed using unique peptides; all unique peptides for the same proteins were taken into account for the comparison of protein abundance. Normalization was conducted using the median intensity of all reporter ions. The ratio (iTRAQ) was determined by comparing with the control (114). We used 1.5-fold change (upregulation) or 0.67-fold change (downregulation) as a cut off for biological significance based on the standard deviation and the normalized peptide ratios.

## Additional files

**Additional file 1.** Experiments on LC-MS analysis.

**Additional file 2.** Raw data for glycopeptides from LC-MS.

**Additional file 3.** Raw data for global peptides from LC-MS.

**Additional file 4.** Regulation of upstream transcriptional regulators on oncogenes and TSGs in lung SqCC and ADC.

**Additional file 5.** Oncogene regulation in SqCC lung tissues.

**Additional file 6.** Oncogene regulation in ADC lung tissues.

**Additional file 7.** Tumor-supressor gene (TSG) regulation in ADC lung tissues.

**Additional file 8.** Tumor-supressor gene (TSG) regulation in SqCC lung tissues.

**Additional file 9.** Identified glycoproteins from ADC and SqCC cancer tissues.

**Additional file 10.** Diseases and biofunctions of lung cancers by ingenuity pathway analysis (IPA).
