# Supplementary material for: Protein signatures of molecular pathways in non-small cell lung carcinoma (NSCLC): comparison of glycoproteomics and global proteomics
Source: Clin Proteomics. 2017 Aug 15;14:31. doi: 10.1186/s12014-017-9166-9 (PMC5557576; doi:10.1186/s12014-017-9166-9)
Supplement: Supplementary file 8 — Additional file 8. Tumor-supressor gene (TSG) regulation in SqCC lung tissues. [file 12014_2017_9166_MOESM8_ESM.docx]

**Supporting Information for**

**Protein signatures of molecular pathways in non-small cell lung carcinoma (NSCLC). Comparison of glycoproteomics with global proteomics**

Shang Yang^1^, Lijun Chen^1^, Punit Shah^1^, Daniel W. Chan, Qing Kay Li^1^*, and Hui Zhang^1*^

^1^ Department of Pathology, Johns Hopkins University, Baltimore, MD 21287

* Address correspondence to

Qing Kay Li MD PhD, Department of Pathology, the Johns Hopkins Medical Institutes, Bayview Medical Center, 4940 Eastern Ave, Baltimore, MD 21244; (o) (410) 550-0671, (f) (410) 550-0075; [qli23@jhmi.edu](mailto:qli23@jhmi.edu).

Hui Zhang PhD, Department of Pathology, the Johns Hopkins Medical Institutes, 4011 Smith Bldg, 400 N Broadway, Baltimore, MD 21287; (o) (410) 502-8149, (f) (410) 502 7882; [hzhang32@jhmi.edu](mailto:hzhang32@jhmi.edu)

**LC-MS Analysis.**

Tryptic peptides or glycopeptides (~1 µg) were separated through a Dionex Ultimate 3000 RSLC nano system (Thermo Scientific) with a 75 µm × 15 cm Acclaim PepMap100 RSLC column (Thermo Scientific) protected by a 5 mm guarding column. The mobile phase flow rate was 350 nL/min using 0.1% formic acid and 2% acetonitrile in water (A) and 0.1% formic acid and 95% acetonitrile in water (B). The gradient profile was set as follows: 5-40% B for 104 min, 40-95% B for 5 min, 95% B for 10 min and equilibrated in 5% B for 15 min. MS analysis was performed using a Thermo Q Exactive mass spectrometer (QE-MS; Thermo Scientific). The spray voltage was set at 1.8 kV. The QE spectra (AGC) target for MS1 was set for 3 × 10^6^ in 60 ms maximum time; AGC target for MS/MS was 5 × 10^4^ (at a resolution of 17,500, intensity threshold of 4 × 10^4^ and maximum IT 100 ms) of the 20 most abundant ions. Charge state screening was enabled to reject unassigned, singly charged, and equal or more than eight protonated ions. A dynamic exclusion time of 25 s was used to discriminate against previously selected ions.

In this study, we analyzed two major subtypes of NSCLC using proteomic and glycoproteomic approaches. Our data demonstrates that expression of proteins and glycoproteins varies in cancer tissues in comparison to tumor-matched benign tissues. More interestingly, our data also demonstrates unique protein signature between SqCC and ADC. Diseases and biofunctions in SqCC and ADC manifest unique characteristics from analysis of protein expression. To evaluate the significance of protein changes in NSCLC, we analyze signaling pathways, upstream regulators, and diseases-biofunctions using IPA. Based on the protein data from each tumor, we find that the majority of canonical pathways are activated on both benign and tumor tissues in SqCC. From NSCLC disease and function analysis, we only observed in benign tissues of ADC subtypes, while ADC tumors show inactivation of those canonical pathways, such as NF-κB, PKA, IL-8 etc. There are other few canonical pathways that have been activated in benign and tumor of SqCC, and in only tumor of ADC. For example, p53 is down-regulated in benign of ADC while it is upregulated in other tissues. Conversely, PPAR and AAVC (antioxidant action of vitamin C) are only activated in ADC but inhibited in SqCC.

The increased protein expression in SqCC or ADC may lead to activation of specific functions in cells, including cell survival, viability , tumor cell viability, tumor growth, tumor cell infection, and cell proliferation. Conversely some diseases and biofunctions are activated in SqCC, whereas they are deactivated in ADC, such as cell metabolism, cell spreading, cell movement, cell invasion, cell internalization, and ROS metabolism. On the other hand, a few of diseases and biofunctions are deactivated in SqCC but are differently regulated in ADC. The results from IPA analysis suggest that organismal death, morbidity or mortality, and tissue tumorigenesis are only triggered in ADC, demonstrating unique activation of these diseases or biofunctions in ADC. Other functions have been deactivated in SqCC and ADC, notably tumor cell apoptosis, cell death, and necrosis. We first discussed proteins based on their functions, followed by pathway analysis.
